# Low Diet Quality and Nutritional Knowledge in Women with Endometriosis: A Pilot Study

**DOI:** 10.3390/healthcare12060673

**Published:** 2024-03-17

**Authors:** Angelika Bogusz, Magdalena Górnicka

**Affiliations:** Department of Human Nutrition, Institute of Human Nutrition Sciences, Warsaw University of Life Sciences, Nowoursynowska St. 159C, 02-776 Warsaw, Poland; angelika_marcinkowska@sggw.edu.pl

**Keywords:** endometriosis, healthy diet, Mediterranean diet, nutritional knowledge, Polish women

## Abstract

Endometriosis is a chronic gynecological disease characterized by ectopic endometrial tissue outside the uterus that can cause chronic pain, dysmenorrhea, fatigue, and infertility. The aim of this study was to assess diet quality and nutritional knowledge of women with endometriosis. A questionnaire was used to collect data on lifestyle, dietary habits and nutritional knowledge from 90 women. More than 80% of women with endometriosis (82.2%) confirmed that diet and lifestyle are related to the progression of endometriosis. After being diagnosed with endometriosis, 77.3% of respondents reported a change in their dietary habits and made various changes to their diet by increasing or eliminating the consumption of certain foods. The majority declared reducing pro-inflammatory products, increasing anti-inflammatory foods, including the consumption of vegetables and fruits, avoiding white/or red/or processed meat, and avoiding sweets. Nevertheless, diet quality as assessed by the pro-healthy diet index (pHDI-10) and the Mediterranean Diet Score (MDS) showed a low intensity of consumption of pro-healthy food (average pHDI-10 value 31.4%; MDS 7.55/14 points, *p* = 0.0001). Women with endometriosis reported lower than recommended consumption of fruits, vegetables, dairy products and whole grains. A higher percentage of healthy controls had a “good” level of nutritional knowledge than women with endometriosis. The results of our study highlight the need to improve diet quality and nutritional knowledge among women with endometriosis as well as developing targeted dietary recommendations to help improve well-being and reduce the pain symptoms of endometriosis.

## 1. Introduction

Endometriosis is an estrogen-dependent, chronic systemic disease with serious reproductive and general health consequences [1], affecting approximately 10% of women of reproductive age worldwide. The classic definition of endometriosis is the presence of endometriotic cell clusters outside the uterine cavity including the pelvic cavity, fallopian tubes, ovaries, upper abdomen, and sigmoid colon [2]. The main symptoms are chronic pain, dyspareunia, dysmenorrhea, dyschezia, dysuria, fatigue, and infertility [3]. The etiology of the disease is complex, multifaceted and not fully understood, but immune, genetic, endocrine, and anatomical abnormalities have been implicated as risk factors [4,5]. Factors contributing to endometriosis include inflammation, oxidative stress, angiogenesis, resistance to apoptosis, and immune disregulation [6]. It has been theorized that increased release of inflammatory cytokines (e.g., prostaglandins, interleukins (IL), tumor necrosis factor (TNF-α) from stromal, epithelial, smooth, or immune cells) contributes to the initiation, development and progression of endometriosis [7]. There are four pathological stages of endometriosis: I—minimal, II—mild, III—moderate, and IV—severe [8]. These stages are differentiated by the advancement of changes in the depth and location of implants as well as the presence and location of adhesions. However, scientific evidence suggests that a correlation between the severity of pain and the stage of endometriosis is rare [9].

The risk of developing endometriosis can be influenced by diet and lifestyle, highlighting the potential impact of diet on the presence of inflammation in the body [10,11,12]. Furthermore, some authors suggest that dietary changes may hold therapeutic promise in mitigating chronic inflammatory processes and reducing visceral pain perception [13]. Specific natural anti-inflammatory agents, such as omega-3 polyunsaturated fatty acids (PUFAs), may reduce inflammation [14] and pain [15].

Dietary factors may be related to physiological processes or symptoms thought to be associated with endometriosis, as they play a role in regulating inflammation, steroid hormone metabolism, prostaglandin levels, and the menstrual cycle, while reducing oxidative stress [10]. In endometriosis, estrogen plays a crucial role in the cellular growth of endometrial tissue [16]. Estrogen can stimulate, facilitate migration, support survival, promote proliferation, and facilitate adherence to the endometrial stroma and epithelial cells [17]. It is produced by the ovaries, skin, adipose tissue and may also be produced locally through a positive feedback loop involving estrogen, prostaglandin E2 (PGE2), aromatase, and cyclooxygenase-2 (COX-2) [18]. Elevated estrogen levels may be another risk factor for the development of endometriosis, an estrogen-dependent condition. Diet has been linked to the development of estrogen-dependent diseases such as endometrial or breast cancer. Diet and nutritional status contribute to the etiology of the disease by influencing blood estrogen levels [19]. Huang et al. showed that dietary factors can influence serum sex-hormone activity and concentrations [20]. Certain dietary fats have the potential to alter endogenous hormone metabolism, and fatty acid intake has been associated with increased levels of endogenous estrogen [21]. One of the dietary strategies is the Mediterranean diet, which can reduce the pain associated with endometriosis due to the recommended products, and therefore the fatty acid profile [22].

In turn, a meta-analysis showed that a high intake of total dairy products may be associated with a reduced risk of endometriosis, whereas a high intake of red meat, saturated fatty acids (SFA) and trans fatty acids (TFA) increase this risk [23]. In addition, some studies suggest that gluten-free and low-nickel diets are associated with improved pain management in endometriosis [24].

However, increasing scientific evidence shows a link between diet, healthy eating and the treatment of endometriosis, especially the reduction in inflammation and pain. Food choices and dietary modifications are undoubtedly also related to nutritional knowledge. Therefore, the aim of the study was to assess the quality of the diet and nutritional knowledge of women with endometriosis.

## 2. Materials and Methods

### 2.1. Participants

The study was conducted in Poland from April to October 2023. In total, 105 people completed the survey, but further analysis included complete data from 90 women (45 with diagnosed endometriosis and 45 without) aged 22–50. Inclusion criteria included female gender, age 18–50 years and consent to research, and for the study group (with endometriosis), at least one year from the diagnosis of endometriosis.

### 2.2. Data Collection

Data were collected via an online survey form that included self-administered questions on lifestyle, questions on the frequency of consumption of selected product groups based on the KomPAN^®^ questionnaire [25] and a 14-item questionnaire on adherence to the Mediterranean diet, which is a repeatable, validated tool [26]. The developed tool was tested in a pilot study on a group of 12 women. The general questionnaire form consists of 4 parts: (1) personal questions (age, height, body weight), health (health status, chronic diseases) and lifestyle (e.g., physical activity); (2) eating habits (e.g., food frequency, snacking); (3) adherence to the Mediterranean diet and (4) nutrition knowledge. In addition, a section with questions specific to women with endometriosis about their medical history and dietary habits was included.

#### 2.2.1. Own Questions

Data were collected on age, anthropometric parameters (height, body mass), medical history (years diagnosed of endometriosis, self-reported chronic disease, prevalence of endometriosis and symptoms of endometriosis), dietary patterns (disease-specific diet, change in diet after diagnosis), lifestyle factors (physical activity) and subjective health assessment. Self-reported questions included body mass, weight and height, which were used to calculate the BMI using the Quetelet equation [body mass (kg)/height (m)^2^] and interpreted according to World Health Organization criteria [27]. Respondents were categorized as underweight (BMI < 18.5 kg/m^2^), normal weight (18.5 kg/m^2^ ≤ BMI < 25.0 kg/m^2^), overweight (25.0 kg/m^2^ ≤ BMI < 30.0 kg/m^2^) and obese (BMI ≥ 30.0 kg/m^2^) [28].

Respondents selected one of three categories to describe their physical activity: 1 as ‘low’; 2 as ‘moderate’; 3 as ‘vigorous’. “Low” physical activity was defined as “1–2 h of physical activity per week”; “moderate” was defined as “2–3 h of physical activity per week”; “vigorous” was defined as “more than 3 h of physical activity per week”.

The data provided were used to create characteristics of the study group and to look for correlations between these and dietary habits and knowledge.

#### 2.2.2. Dietary Habits and Diet Quality

The study used a Food Frequency Questionnaire (FFQ) KomPAN^®^ validated for the Polish population [25]. The form included questions on food groups such as vegetables, fruits, whole grain bread, white bread, whole grain cereals, oatmeal, whole grain pasta, white rice, noodles, finely ground groats, nuts, legumes, fish, eggs, vegetable oils, butter, white and red meat, milk and dairy products, fried dishes, fast food, and water. Six categories of frequency of consumption were used (1—never, 2—1–3 times a month, 3—once a week, 4—several times a week, 5—every day; 6—several times a day).

Respondents also provided information about special diets, eating meals at regular times and number of meals per day, and snacking between meals.

To assess overall diet quality, a pro-healthy diet index (pHDI-10) score was calculated based on previous knowledge and other validated studies [25]. The questionnaire used closed-ended questions on the frequency of consumption of 10 food groups (whole grain bread; whole grain cereals, oatmeal, whole grain pasta; milk; fermented milk drinks; cottage cheese; white meat; fish; dishes with legumes; fruit; vegetables). For each food item, the frequency consumption categories were converted into values reflecting the daily frequency of consumption: (1) never = 0.00, (2) 1–2 times/month = 0.06, (3) once a week = 0.14, (4) 2–3 times/week = 0.5, (5) once a day = 1 and (6) several times a day = 2. The pHDI-10 was expressed in percentage points and categorized as follows: low (0–33.32% points), moderate (33.33–66.65% points) and high (66.66–100% points) intensity of consumption of selected pro-healthy food groups.

##### Adherence to Mediterranean Diet

The 14-item Mediterranean Diet Adherence Screener (MEDAS) questionnaire was used to assess diet quality [26]. The questionnaire included dietary components such as olive oil consumption, meat preferences, nut consumption, fizzy drinks, and Mediterranean sauce consumption. The MEDAS questionnaire, originally developed in a Spanish case–control study, consists of 14 items assessing adherence to the Mediterranean diet. Nine items were initially selected on the basis of cut-off points that distinguished cases from controls. Subsequently, five additional items relevant to the assessment of the traditional Mediterranean diet were included. The questionnaire covers dietary habits related to olive oil, meat preferences, nut consumption, fizzy drinks, and the use of a Mediterranean sauce called “sofrito”. Adherence to the Mediterranean diet was expressed as the Mediterranean Diet Score (MDS). Scores on the MEDAS questionnaire were assigned based on participants’ responses, with a total possible score ranging from 0 to 14. Higher scores indicated greater adherence to the Mediterranean diet.

#### 2.2.3. Nutrition Knowledge

Nutrition knowledge was also assessed using the KomPAN^®^ validated questionnaire. The questionnaire consisted of 26 nutrition knowledge questions to which respondents answered ‘true’, ‘false’, or ‘hard to say’. For each correct answer, the respondent received 1 point. Respondents could score a maximum of 26 points. After reviewing and calculating the total score, respondents were given an overall knowledge score. Respondents who scored between 0 and 9 points were considered to have insufficient knowledge, those who scored between 10 and 17 points were considered to have sufficient knowledge and those who scored between 18 and 26 points were considered to have good nutrition knowledge [29].

### 2.3. Statistical Analysis

The analyses were carried out using the STATISTICA 13.3 package (StatSoft Inc., Tulsa, OK, USA) under the license of the Warsaw University of Life Sciences. After completion of the online survey and data collection, the final database was downloaded as a Microsoft Excel (Microsoft Office 16, 2019) spreadsheet and subjected to data analysis. Open-ended questions were carefully reviewed, condensed, and coded for statistical analysis. Means, SDs, and percentages were calculated to represent the data. The Chi squared test (χ^2^) test was used to examine differences between categorical variables. Because non-parametric data were obtained, for the relationships between two independent variables, the Mann–Whitney U test was used to determine statistically significant differences between the two groups. Statistical significance was considered at *p* < 0.05.

## 3. Results

### 3.1. Characteristics of Study Group

The study group consisted of 90 women, including 45 women with endometriosis and 45 women without endometriosis. Baseline characteristics of the study group are shown in Table 1. Women with endometriosis were younger than non-endometriosis women (29.97 ± 7.06 vs. 33.13 ± 5.85, *p* = 0.007). The mean BMI was not significantly different between the groups. More women with endometriosis reported poorer subjective health (35.6% vs. 4.4%, *p* = 0.001). There were no significant differences in physical activity between the groups.

**Figure 1:**
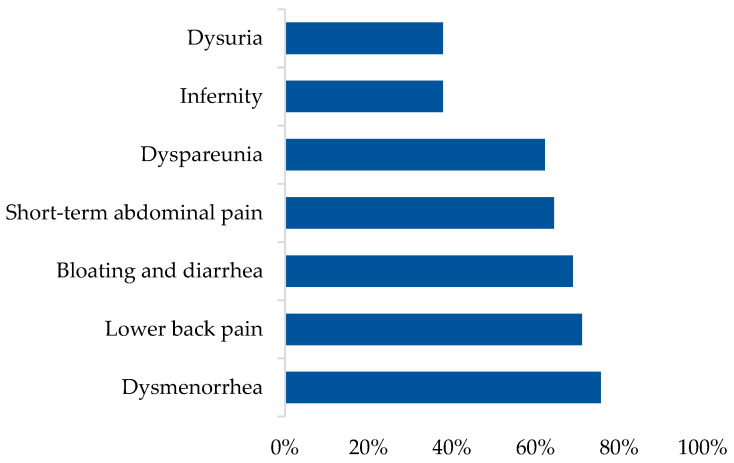
The symptoms of endometriosis.

In the endometriosis group (*n* = 45), women reported dysmenorrhea (75.6%), lower back pain (71.1%), bloating and diarrhea (68.9%), short-term abdominal pain (64.4%), dyspareunia (62.2%), infertility (37.8%), and dysuria (37.8%) as major symptoms (Figure 1).

**Figure 2:**
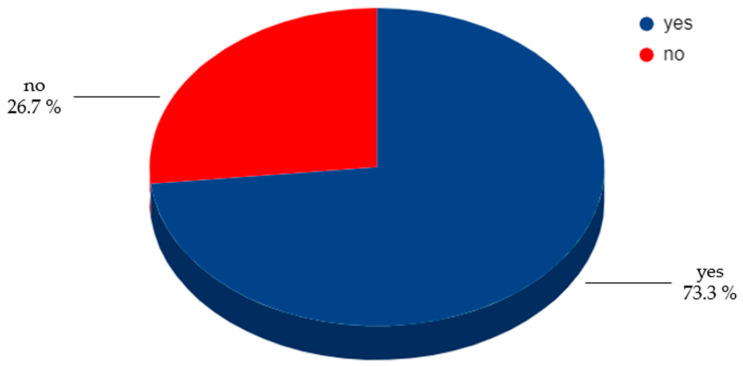
Changes in dietary habits after diagnosis of endometriosis.

More than 80% of women with endometriosis (82.2%) confirmed that diet and lifestyle are related to the progression endometriosis. After the diagnosis of endometriosis, 77.3% of respondents reported a change in their dietary habits (Figure 2).

**Figure 3:**
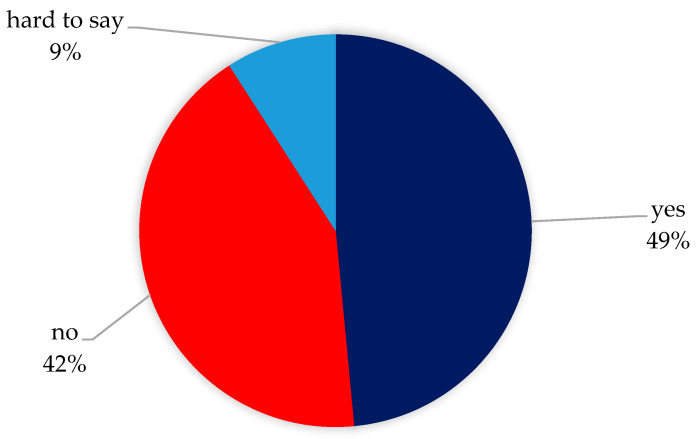
Improvement in well-being after implementing the diet.

Almost half of the women who followed diet reported an improvement in well-being after implementation (Figure 3).

**Figure 4:**
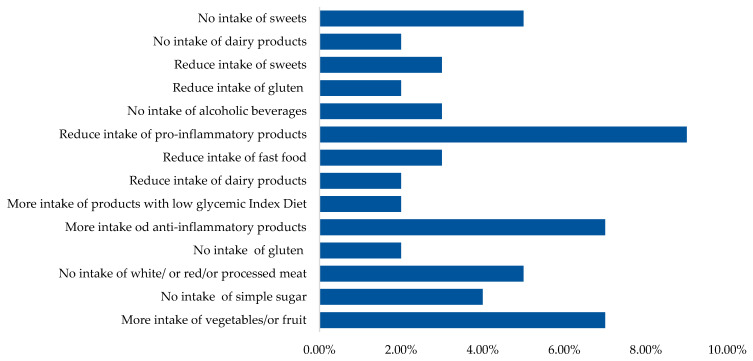
Food choices of women after diagnosis of endometriosis.

After being diagnosed with endometriosis, women made various changes to their diet by increasing or eliminating the consumption of certain foods (Figure 4). The majority declared reducing pro-inflammatory products, increasing anti-inflammatory foods, including the consumption of vegetables and fruits, avoiding white/or red/or processed meat, and avoiding sweets.

**Figure 5:**
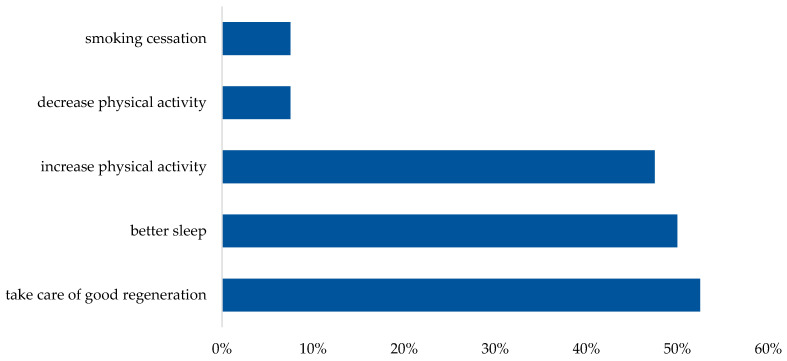
Changes in lifestyle after diagnosis of endometriosis.

Women with endometriosis reported a change in lifestyle (Figure 5) after diagnosis by ensuring good regeneration (52.5%), better sleep (50%) and increased physical activity (47.5%). Most women use dietary supplements (77.8%) such as vitamin D, magnesium, omega-3 fatty acids, ashwagandha or coenzyme Q10.

### 3.2. Frequency of Consumption of Selected Product Groups and Dietary Guidelines

Most women declared eating 3–4 meals, eating regularly, and snacking (Table 2). Women without endometriosis were likely to report eating 3–4 meals (80% vs. 69%; (*p* = 0.03) and more likely to report snacking (several times a week) (Table 2).

**Figure 6:**
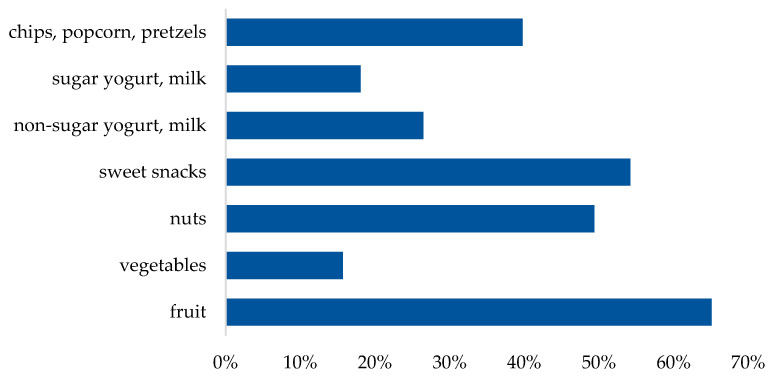
Snacks between meals.

The most commonly reported snack category was fruit, sweets and nuts (Figure 6).

The comparison of the frequency of consumption of selected foods in the group with endometriosis and in the group without endometriosis is presented in Table 3. The mean frequency of consumption of fruits and vegetables in the group was lower than recommended (several times a day_ and the frequency of consumption of dairy products, whole grains, and fish was different from the recommendations. The frequency of consumption of red and processed meat was high and above dietary recommendations. Women with endometriosis declared more frequent consumption of nuts, legumes, and fish, but less frequent consumption of white rice, white bread, pasta, vegetable oils, milk, cheese, fried dishes and fast food than women without endometriosis (*p* ≤ 0.05).

### 3.3. Diet Quality

As shown in Table 4, both groups had a low intensity of consumption of pro-healthy foods.

Women with endometriosis were statistically more likely to follow the Mediterranean diet. Women with endometriosis ate more fish and dishes with legumes and nuts, which were the main factors involved in increasing MDS. However, the consumption of vegetables and fruits was insufficient.

### 3.4. Nutritional Knowledge

Among the respondents, 52 individuals (57.8%) demonstrated a ‘good’ level of nutritional knowledge. The level of nutritional knowledge depended significantly on the prevalence of endometriosis (*p* < 0.047) (Table 5). A higher percentage of women without endometriosis than women with endometriosis were characterized by a ‘good’ level of nutritional knowledge. There was no significant difference in the level of nutritional knowledge according to MDS compliance and pHDI-10. The least correct answers were given to the question ‘Protein should be the main source of energy in a proper diet’.

## 4. Discussion

Our results showed that diet quality was poor in the whole group. The frequency of consumption of healthy and unhealthy foods did not match the recommendations. Our study found that the frequency of eating healthy foods such as fruits, vegetables, dairy products and whole grains was low, although women with endometriosis reported eating nuts, legumes and fish more often than women without endometriosis. This is visible in the higher MDS value in this group compared to the control group. In the study group, almost 60% had “good” nutritional knowledge, and these were more often women without endometriosis.

Diet may be involved in the progression and severity of endometriosis [30]. According to the number of studies, certain dietary components, including vegetables, fruits, dairy products and omega-3 PUFAs, have been associated with a lower risk of developing endometriosis [10,31,32], while dietary factors such as fiber, antioxidants, vitamin D and B groups may have helpful effects for prevention and treatment. However, while some studies have noted observations supporting the importance of dietary interventions, only a small fraction have provided evidence-based dietary recommendations [33]. Given the complexity of the disease, this is not surprising [34]. The general recommendation is to consume a diet high in fiber, with an emphasis on fruits and vegetables, while minimizing the intake of sugar and animal products [35].

Understanding the underlying mechanisms, such as anti-inflammatory properties and hormonal modulation, is crucial for developing targeted dietary interventions and advancing our knowledge of endometriosis prevention. Some studies have shown that a higher consumption of fruits and green vegetables may be protective because it can reduce inflammatory markers such as IL-6 [36], which are thought to be involved in the pathogenesis of endometriosis. In addition, a plant-based diet decreases the bioavailability of estrogen. Thus, estrogen plays a role in inducing the proliferation of extra-uterine endometrial tissue [37]. Evidence suggests that a high dairy consumption during adolescence may be associated with a reduced risk of developing endometriosis later in life. Zemel et al. found that increased calcium and dairy intake led to a reduction in inflammatory factors such as TNFα and IL-6 and oxidative stress such as reactive oxygen species (ROS) [38], which are the main hallmarks of endometriosis [39]. PUFAs, such as eicosapentaenoic acid (EPA) and docosahexaenoic acid (DHA), which are derived from fish, may reduce inflammation [14]. However, PUFA omega-3 has been shown to play a role in regulating and reducing inflammatory prostaglandins and cytokines (IL-1, 2, and 6, TNF-α) [40]. Moreover, women with endometriosis reported a lower frequency of eating foods such as white rice, white bread, pasta, cheese, fried dishes and fast food than women without endometriosis. A high intake of SFAs including animal products such as cheese and a high consumption of products containing TFA including fast foods were linked with a higher risk of developing endometriosis [11]. The pro-healthy quality of the diet in the study group was not satisfactory (low pHDI-10). It was found that fish, dishes with legumes and cottage cheese intake were the main factors involved in lowering the pHDI-10. This is consistent with the results of an observational study in which Dutch women with endometriosis had a slightly lower diet quality (Dutch Healthy Diet Index 2015) than healthy women [41].

Armour et al. showed that 76% of women with endometriosis use self-treatment strategies, including self-care or lifestyle changes, including diet. This confirms the significant impact of the disease on women’s perceptions of diet and health [42]. These results are consistent with our findings that 77.3% of the women with endometriosis made dietary changes. Also, Mazza et al. reported that 66.4% of women surveyed changed their diet after being diagnosed with endometriosis, suggesting that women are exploring a variety of dietary strategies, such as gluten-free, anti-inflammatory, Mediterranean, or ketogenic diets, to alleviate endometriosis-related symptoms and improve quality of life. In addition, this study showed that an anti-inflammatory diet and elimination of foods rich in SFAs and simple sugars were more commonly used by women with severe disease [43]. In our study, women with endometriosis most often reported limiting the consumption of pro-inflammatory products, increasing the consumption of anti-inflammatory foods, increasing the consumption of vegetables and fruits, and avoiding the consumption of white/or red/processed meat and sweets. This is undoubtedly related to the attempt to treat the inflammation associated with endometriosis.

Research has shown that endometriosis is initiated through mechanisms involving inflammation, including cell proliferation and infiltration. Macrophages play a pivotal role in endometriosis, and their activation, coupled with increased cytokine secretion, significantly influences lesion progression, proliferation, and angiogenesis. Proinflammatory cytokines, including IL such as IL-1, IL-8, IL-33, nuclear factor kappa B (NF-κB), and TNFα, have been widely documented during various stages of endometriosis progression [44]. Thus, nutrients with anti-inflammatory properties have the potential to alleviate pain symptoms associated with endometriosis [45]. Simultaneously, increased adherence to an anti-inflammatory diet may indicate a response to the severity of symptoms and the need to address inflammatory processes.

One of the dietary strategies with anti-inflammatory effects is the Mediterranean diet, which is characterized by the use of olive oil as the primary source of fat, coupled with an increased intake of fruits, nuts, vegetables, legumes, whole grains, spices, and herbs. It also includes a moderate consumption of dairy products, with an emphasis on cheese and yogurt, along with fish and poultry. Conversely, the diet reduces the consumption of red meat and processed foods [46]. The Mediterranean diet may reduce pain associated with endometriosis through some mechanisms. First, some products such as olive oil and fish have been shown to have anti-inflammatory effects [22]. Olive oil has been shown to reduce markers of inflammation such as IL-6 and TNFα [46]. Second, oleocanthal, found in olive oil, is associated with cyclooxygenase inhibition through a mechanism similar to ibuprofen [47]. Moreover, various spices are often included in the Mediterranean diet and anti-inflammatory patterns. Recent studies have provided evidence for the efficacy of these spices and their bioactive compounds in preventing and alleviating various chronic diseases, including arthritis, asthma, cancer, neurodegenerative disorders [48,49]. The results of the study by Ott et al. confirmed the effect of the Mediterranean diet in reducing pain associated with endometriosis. Remarkable pain relief, including improvements in overall pain, dysmenorrhea, dyschezia, dyspareunia, and general well-being, was observed in women who followed a specific diet that included fruits, fresh vegetables, fatty fish, white meat, legumes, whole grains, magnesium-rich foods, and olive oil [50]. In our study, women with endometriosis had a statistically higher adherence to the Mediterranean diet than women without endometriosis. The frequency of consumption of nuts, fish, and dishes with legumes was higher in women with endometriosis compared to those without the condition, leading to an increase in MDS. Moreover, women with endometriosis declared various dietary changes, including increasing or eliminating the consumption of certain foods after being diagnosed with endometriosis. The majority declared reducing the consumption of pro-inflammatory products, increasing the consumption of anti-inflammatory foods, and increasing the consumption of vegetables and fruits, not eating white/or red/or processed meat, and not eating sweets. Haaps et al. showed that Dutch women who strictly adhered to the endometriosis diet recommended by the Dutch Endometriosis Society, based on the avoidance of red meat, products containing gluten, cow’s milk, sugars (refined, natural sugars and sweeteners), foods high in estrogen (soy, tofu, soya sprout, miso, tempeh, linseed, sesame seeds) and limited caffeine (max 200 mg daily), reported a significantly improved quality of life (QoL) [51]. Foods high in phytoestrogens (PEs) have been identified in various types of plant foods. PEs have a structure similar to estrogens with a phenolic ring and two hydroxyl groups, which allows them to act as weak estrogenic agents and disrupt hormonal and molecular signaling. In addition, phytoestrogens may exhibit weak estrogenic activity in low estrogen environments such as menopause, while demonstrating antiestrogenic effects in high estrogen environments, such as those found in endometriosis [16]. However, the effect of phytoestrogens on the risk of developing and progression of endometriosis remains unclear [16,52,53].

The results of the prospective study highlighted the positive impact of a low-FODMAP (FODMAP—Fermentable Oligo-, Di-, Monosaccharides And Polyols) diet on both the pain scores and QoL in women with endometriosis [54] who very often reported more abdominal discomfort and pain, constipation, bloating, flatulence, and bowel urgency than healthy women [55]. These non-specific complaints, such as gastrointestinal symptoms, overlap with other chronic conditions that reduce quality of life, such as irritable bowel syndrome (IBS), inflammatory bowel disease (IBD) and celiac disease (CD) [56]. Dietary strategies recommended for these conditions, such as low-FODMAP [57], gluten-free [58], and low-nickel diets [59], have been shown to improve symptoms in women diagnosed with endometriosis. In our study, less than half of the participants reported an improvement in well-being after implementing the diet.

Dietary guidelines for endometriosis are largely based on limited evidence, although research suggests that dietary changes are often a self-management strategy. Women with endometriosis search for dietary methods to improve their well-being. They often use elimination diets, regularly excluding various foods such as those containing gluten or lactose, without appropriate substitutes, which can lead to nutritional deficiencies [60]. However, the effectiveness of specific dietary interventions for women with different types of endometriosis or individual characteristics remains unclear [43].

In the absence of clear recommendations for women with endometriosis, they often turn to social media for support. An analysis of endometriosis-related content available on Instagram found that only four categories (medicine, research articles, symptoms and fertility) contained claims that were at least 50% evidence-based. Conversely, many of the claims in different categories had varying degrees of evidence-based, mixed, and non-evidence-based information. The implications of this are significant, given the potential impact of social media on the decision-making and management of women with endometriosis [61].

Apart from the unsatisfactory quality of the diet in the endometriosis group, the level of nutritional knowledge was sufficient or good. Moreover, our study showed that women with endometriosis had lower nutritional knowledge than the controls. No differences were found in the relationship between nutritional knowledge and adherence to the Mediterranean diet, pro-healthy diet and BMI in women with endometriosis. To our knowledge, no studies have been conducted between nutritional knowledge and diet quality in women with endometriosis, but these results are similar to those of studies performed by Bykowska-Derda et al. who analyzed dietary habits identified by diet quality scores in the context of body fatness and nutritional knowledge in women with polycystic ovary syndrome (PCOS). PCOS is a common female endocrinopathy that is recognized as a heterogeneous disorder. This study showed that the nutritional knowledge of PCOS women was lower than the healthy controls. Also, the PCOS group had a significantly lower frequency of consumption of pro-healthy foods (pHDI-10) [62].

The strength of our study is the relatively large amount of data collected in the study. We presented multiple results, including health and lifestyle data, anthropometric data, dietary habits, adherence to the Mediterranean diet, diet quality, and nutritional knowledge. In addition, FFQ, diet quality (pHDI-10) and adherence to the Mediterranean diet (MDS) were based on the validated questionnaire, which is also a strength of this study. Moreover, as the forms used for the study were distributed online, respondents could complete them anywhere at any time. Although the study achieved its objectives, it may have some limitations. First, the study sample is not representative of the general population, but included women who voluntarily submitted the online form. The generalizability of the findings may be limited to the specific population studied. Different demographic, ethnic, or cultural groups may have differences in dietary habits and their associations with endometriosis. Second, dietary information was collected through self-report surveys, which are susceptible to social desirability bias. Participants may provide answers that conform to perceived societal expectations rather than accurately reflecting their actual dietary behaviors. The cross-sectional design of the study also limits the ability to establish causal relationships between dietary habits, nutritional knowledge, and endometriosis. It provides only a snapshot of the relationship at one point in time, preventing the assessment of temporal relationships or changes over time. Despite the limitations, the results obtained have research potential and practical significance. Although the results provide valuable insights into the dietary habits, diet quality and nutritional knowledge of women with and without endometriosis in the sample studied, they may not be universally applicable to all women with this disease. Therefore, further research using larger, more diverse samples and longitudinal designs is needed to validate and extend the findings of this study, thereby increasing their generalizability to broader populations of women with endometriosis.

**Figure 7:**
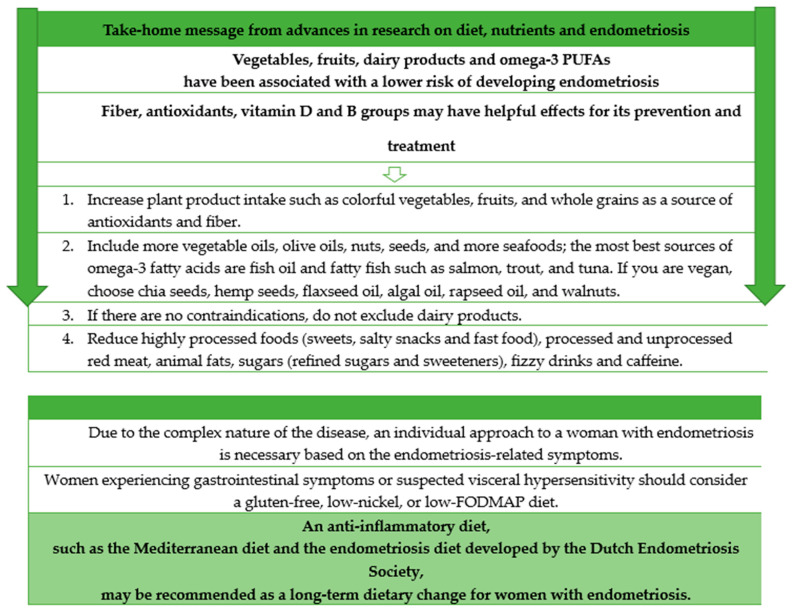
Take-home message from advances in the research on diet, nutrients and endometriosis.

A summary of our own and other authors’ results [11,32,51,58,63,64,65] for potential dietary modifications in the case of endometriosis is presented below (Figure 7).

## 5. Conclusions

The results of our study highlight the need to improve diet quality and nutritional knowledge among women with endometriosis. Women with endometriosis reported various changes to their diet, but the overall health-promoting quality of the diet was low. Self-management of diet in women with endometriosis may result in a deterioration of the quality of their diet and no noticeable impact on the improvement of well-being. The results of our study highlight the importance of nutritional counseling, dietary interventions, and the development of dietary guidelines, which may be important in reducing endometriosis pain symptoms.

Further research is needed to obtain evidence on the specific impact of nutrients on endometriosis activity and, consequently, to develop personalized nutritional strategies tailored to the unique needs and situations of women with endometriosis.

## Figures and Tables

**Table 1:** Baseline characteristics of the participants.

Variables	Total	Endometriosis *n* = 45	Non-Endometriosis *n* = 45	*p*-Value *
	Mean ± SD	Mean ± SD	Mean ± SD	
Age [y]	31.55 ± 6.60	33.13 ± 5.78	29.98 ± 6.98	0.007
Height [cm]	165.96 ± 6.51	165.57 ± 6.88	166.33 ± 6.10	ns
Body mass [kg]	60.52 ± 10.51	59.60 ± 10.81	61.44 ± 10.12	ns
BMI (kg/m^2^)	21.96 ± 3.55	21.67 ± 3.21	22.26 ± 3.85	ns
Self-reported health status	[*n*, %]			
very good	14 (15.6%)	3 (6.7%)	11 (24.4%)	0.001
good	30 (33.3%)	7 (15.6%)	23 (51.1%)	
fair	28 (31.1%)	19 (42.2%)	9 (20.0%)	
hard to say	8 (8.9%)	7 (15.6%)	1 (2.2%)	
poor	10 (11.1%)	9 (20.0%)	1 (2.2%)	
Physical activity	[*n*, %]			
low	53 (58.9%)	25 (55.5%)	28 (62.2%)	0.563
moderate	25 (27.8%)	13 (28.9%)	12 (26.7%)	
vigorous	12 (13.3%)	7 (15.6%)	5 (11.1%)	

* Chi squared test; BMI—Body Mass Index; ns—not significant; SD—standard deviation.

**Table 2:** Dietary habits of endometriosis and non-endometriosis.

Dietary Habits	Total *n*, %	Endometriosis *n*, %	Non-Endometriosis *n*, %	*p*-Value *
Number of meals				
1–2	3 (3.3%)	0	3 (6.7%)	0.03
3–4	67 (74.5%)	31 (68.9%)	36 (80%)	
5	20 (22.2%)	14 (31.1%)	6 (13.3%)	
Eating regularly				
Yes	82 (91.1%)	41 (91.1%)	41 (91.1%)	ns
No	8 (8.9%)	4 (8.9%)	4 (8.9%)	
Snacking				
Yes	82 (91.1%)	39 (91.1%)	43 (95.6%)	ns
No	8 (8.9%)	6 (8.9%)	2 (4.4%)	
Frequency of eating snacks				
several times a day	6 (7.3%)	2 (5.1%)	4 (9.3%)	*p* < 0.001
once a day	11 (13.4%)	3 (7.7%)	8 (18.6%)	
several times a week	36 (44.0%)	14 (35.9%)	22 (51.2%)	
once a week	18 (21.9%)	12 (30.8%)	6 (14.0%)	
1–3 times a month	11 (13.4%)	8 (20.5%)	3 (6.9%)	

* Chi squared test; ns—not significant.

**Table 3:** The comparison of the mean frequency of consumption of selected food products and drink in the endometriosis group and the non-endometriosis group.

Food Groups	Total *n* = 90	Endometriosis *n* = 45	Non-Endometriosis *n* = 45	*p*-Value *
	Mean ± SD Median	Mean ± SD Median	Mean ± SD Median	
Vegetables	5.54 ± 0.96	5.62 ± 0.82	5.46 ± 1.07	ns
	6	6	6	
Fruits	5.26 ± 1.04	5.33 ± 1.05	5.18 ± 1.02	ns
	5	6	5	
Whole grain bread	3.77 ± 1.57	3.77 ± 1.81	3.77 ± 1.27	ns
	4	4	4	
White bread	3.50 ± 1.60	3.07 ± 1.74	3.93 ± 1.32	0.015
	4	3	4	
Whole grain cereals, oatmeal, whole grain pasta	3.54 ± 1.26	3.80 ± 1.30	3.29 ± 1.17	ns
	4	4	4	
White rice, noodles, finely ground groats	3.17 ± 1.03	2.84 ± 1.02	3.49 ± 0.93	0.007
	3	3	4	
Nuts	2.53 ± 1.15	3.00 ± 1.03	2.07 ± 1.06	0.0002
	3	3	3	
Legumes	2.87 ± 1.27	3.13 ± 1.17	2.59 ± 1.30	0.032
	3	3	2	
Fish	2.46 ± 0.92	2.76 ± 0.95	2.18 ± 0.79	0.006
	2	3	2	
Eggs	3.63 ± 1.12	3.82 ± 1.12	3.44 ± 1.09	ns
	4	4	4	
Vegetable oils	3.68 ± 1.53	3.22 ± 1.69	4.13 ± 1.20	0.016
	4	4	4	
Butter	3.34 ± 1.80	3.39 ± 1.88	3.29 ± 1.73	ns
	3	3	3	
Red meat	2.36 ± 1.20	2.18 ± 1.08	2.53 ± 1.28	ns
	2	2	3	
White meat	3.26 ± 1.39	3.44 ± 1.40	3.07 ± 1.36	ns
	4	4	4	
Milk	3.27 ± 1.76	2.84 ± 1.73	3.80 ± 1.61	0.013
	4	3	4	
Fermented milk drinks	3.44 ± 1.38	3.16 ± 1.49	3.71 ± 1.20	ns
	4	3	4	
Cottage cheese	2.73 ± 1.37	2.47 ± 1.34	3.00 ± 1.35	ns
	4	3	4	
Cheese	3.11 ± 1.40	2.55 ± 1.34	3.67 ± 1.23	0.0001
	3	2	4	
Fried dishes	3.11 ± 1.06	2.84 ± 1.07	3.39 ± 0.98	0.026
	3	3	4	
Fast food products	2.0 ± 0.7	1.82 ± 0.7	2.18 ± 0.64	0.014
	2	2	2	
Water	5.86 ± 0.6	5.93 ± 0.33	5.78 ± 0.79	ns
	6	6	6	

* The Mann–Whitney U test; SD—standard deviation, ns—not significant. Frequency categories: 1—never, 2—1–3 times a month, 3—once a week, 4—several times a week, 5—every day; 6—several times a day.

**Table 4:** Dietary indexes in endometriosis and non-endometriosis group.

Diet Indexes	Total *n* = 90	Endometriosis *n* = 45	Non-Endometriosis *n* = 45	*p*-Value *
	Mean ± SD Median	Mean ± SD Median	Mean ± SD Median	
pHDI-10 (%)	31.4 ± 10.64	32.64 ± 11.47	30.1 ± 9.57	ns
	29.75	30	29.50	
MDS (points)	6.82 ± 1.78	7.55 ± 1.80	6.09 ± 1.44	0.0001
	7	8	6	

* The Mann–Whitney U test; SD—standard deviation; ns—not significant; pHDI-10—pro-healthy diet index; MDS—Mediterranean Diet Score.

**Table 5:** Nutritional knowledge level in the study group.

Variables	Nutritional Knowledge Level			*p*-Value *
	Insufficient	Sufficient	Good	
Total group (*n*, %)	2 (2.2%)	36 (40%)	52 (57.8%)	**-**
Endometriosis (*n*, %)				
Yes	1 (1.1%)	21 (46.7%)	23 (51.1%)	*p* = 0.047
No	1 (2.2%)	15 (33.3%)	29 (64.4%)	
MDS				ns
≤7	2 (100%)	24 (66.7%)	30 (57.7%)	
8–9	0	10 (27.8%)	17 (32.7%)	
≥10	0	2 (5.56%)	5 (9.6%)	
pHDI-10 (*n*, %)				ns
low	0	32 (88.9%)	40 (76. 9%)	
moderate	0	4 (11.1%)	12 (23.1%)	
high	0	0	0	
BMI (kg/m^2^)	21.40 ± 0.83	22.67 ± 4.08	21.50 ± 3.13	ns
Age	37 ± 2	31.95 ± 6.26	32.14 ± 6.49	ns

* The Mann–Whitney U test; ns—not significant; pHDI-10—pro-healthy diet index; MDS—Mediterranean Diet Score; BMI—Body Mass Index.

## Data Availability

These data are available from the first author.
