# Correction: Bogusz, A.; Górnicka, M. Low Diet Quality and Nutritional Knowledge in Women with Endometriosis: A Pilot Study. *Healthcare* 2024, *12*, 673

**DOI:** 10.3390/healthcare12100973

**Published:** 2024-05-09

**Authors:** Angelika Bogusz, Magdalena Górnicka

**Affiliations:** Department of Human Nutrition, Institute of Human Nutrition Sciences, Warsaw University of Life Sciences, Nowoursynowska St. 159C, 02-776 Warsaw, Poland; angelika_marcinkowska@sggw.edu.pl

## References

The authors would like to make the following corrections to the publication, dealing with references #25 and #42 [1].

We suspect that a typo in one of the drafts resulted in a wrong citation.

25.Stasiewicz, B. *Dietary Habits and Nutrition Beliefs Questionnaire and the Manual for Developing of Nutritional Data KomPAN (Kwestionariusz do Badania Poglądów i Zwyczajów Żywieniowych Oraz Procedura Opracowania Danych (KomPAN^®^): Wersja Polskojęzyczna)*; Committee of Human Nutrition Science, Polish Academy of Science: Warsaw, Poland, 2020.

Should be revised to:

25.Jeżewska-Zychowicz, M.; Gawęcki, J.; Wądołowska, L.; Czarnocińska, J.; Galiński, G.; Kołłajtis-Dołowy, A.; Roszkowski, W.; Wawrzyniak, A.; Przybyłowicz, K.; Krusińska, B.; et al. Dietary Habits and Nutrition Beliefs Questionnaire for people aged 16 to 65, version 1.1. In *Dietary Habits and Nutrition Beliefs Questionnaire and the Manual for Developing of Nutritional Data (In Polish: Kwestionariusz do Badania Pogladów i Zwyczajów Żywieniowych oraz Procedura Opracowania Danych (KomPAN^®^))*; Gawęcki, J., Ed.; Committee of Human Nutrition Science, Polish Academy of Science: Warsaw, Poland, 2014; pp. 3–20. Available online: http://www.knozc.pan.pl/ (accessed on 5 February 2023).

The author mistakenly added a wrong reference during proofreading.

42.Armour, R.; Howes, R.F. *Coleridge the Talker: A Series of Contemporary Descriptions and Comments*; Cornell University Press: Ithaca, NY, USA, 2019.

Should be revised to:

42.Armour, M.; Sinclair, J.; Chalmers, K.J.; Smith, C.A. Self-management strategies amongst Australian women with endometriosis: A national online survey. *BMC Complement. Altern. Med.*
**2019**, *19*, 17. https://doi.org/10.1186/s12906-019-2431-x.

The authors apologize for any inconvenience caused and state that the scientific conclusions are unaffected. This correction was approved by the Academic Editor. The original publication has also been updated.
